# A text data mining approach to the study of emotions triggered by new advertising formats during the COVID-19 pandemic

**DOI:** 10.1007/s11135-022-01460-3

**Published:** 2022-06-30

**Authors:** Angela Maria D’Uggento, Albino Biafora, Fabio Manca, Claudia Marin, Massimo Bilancia

**Affiliations:** 1 Department of Economics and Finance (DIEF), University of Bari Aldo Moro, Largo Abbazia di S. Scolastica, 70124 Bari, Italy; 2 Master’s Degree Programme in Data Science, Department of Computer Science (DIB), University of Bari Aldo Moro, Via E. Orabona 4, 70125 Bari, Italy; 3 Department of Education, Psychology, Communication (ForPsiCom), University of Bari Aldo Moro, Palazzo-Chiaia Napolitano, Via Scipione Crisanzio 42, 70124 Bari, Italy; 4 Ionic Department (DJSGEM), University of Bari Aldo Moro, Via Duomo 259, 74123 Taranto, Italy

**Keywords:** COVID-19 pandemic, Twitter, TV spots, Text mining, Mixture of Unigrams

## Abstract

Under the influence of the health emergency triggered by the COVID-19 pandemic, many brands changed their communication strategy and included more or less explicit references to the principles of solidarity and fraternity in their TV commercials to boost the confidence and hope of Italian families during the lockdown. The traditional attitudes of the advertising format, which focused on product characteristics, were relegated to the background in order to reinforce the “brand image” through words, signs, hashtags and music that spread empathetic messages to all those who needed to regain hope and trust in a time of extreme emotional fragility. The objective of this paper is to identify the emotions and brand awareness during the lockdown using text mining techniques by measuring customer sentiment expressed on the Twitter social network. Our proposal starts from an unstructured corpus of 20,982 tweets processed with text data mining techniques to identify patterns and trends in people’s posts related to specific hashtags and TV ads produced during the COVID-19 pandemic. The innovations in the brand’s advertising among consumers seem to have triggered some sense of appreciation and gratitude, as well as a strong sense of belonging that was not present before, as the TV ads were perceived as a disruptive element in consumers’ tweets. Although this effect is clearly documented, in this paper we demonstrate its transitory nature, in the sense that the frequency of occurrence of terms associated with an emotional dimension peaks during the weeks of lockdown, and then gradually decreases.

## Introduction

In 2020, the world was struck by an unknown pandemic, with completely unexpected and devastating effects that continue to this day. Individuals and communities experienced radical changes in lifestyle, organization of work, study and leisure. The COVID-19 pandemic shocked the world and led to a global lockdown in March 2020 and has since reemerged in a second and third wave in several parts of the world (Liu et al. 2020). The restrictive measures introduced during the first wave forcibly kept people at home, causing them to reorganize their daily routines, adapt their habits to the new situation, and change perceptions of what was previously considered “normal”.

From an economic point of view, all sectors have suffered severe losses, especially the sales of sectors related to the free mobility of customers, such as tourism, travel, catering, which have collapsed; the only exceptions were food, health and e-commerce. The impact of the pandemic varied widely by industry (e.g., airlines vs. flour manufacturers), medium (e.g., digital media vs. magazines), and primary market served (Taylor 2020). As a “sensor” always ready to perceive changes in society, advertising also adapted to the new mood created by the pandemic and looked for a new way to address the public to cope with the sales crisis (Gangadharbatla 2021). In general, companies used different strategies depending on the type of goods sold, but in the vast majority of cases they focused on two main objectives: respecting the sensitivity of a large number of people affected by the sudden loss of a loved one, on the one hand, and growing empathy with consumers, on the other. There have been situations where well-known brands have decided to withdraw their commercials: this is the case of KFC, which released a “finger-licking” commercial for British TV in late February and withdrew it just two weeks later because it was deemed inappropriate.

Social distancing and appropriate behaviour have suddenly become guidelines for institutional communication, along with hashtags to alert people around the world to the most important restrictive measure to contain the contagion: staying home. #IoRestoACasa, #IStayAtHome, #YoMeQuedoEnCasa, #JesuisChezMoi suddenly became the most popular hashtags in the 3 media (print, broadcast, and Internet), and brands adopted them in their advertising, along with assurances of continued availability of food and other goods to meet basic needs. These messages quickly became necessary after the wild hoarding of basic goods that characterised the initial phase of the pandemic, and companies immediately began to insure the continuous availability of supplies. For example, the Spanish insurance company Assist Card invited people to be “infected”—alluding to the meaning of the word—by those who did not fill their shopping carts, did not raise prices to speculate, from those who stayed at home. In the Italian edition of the commercial there were many images of balconies, those of the first days, with waving national flags and the Italian anthem in the background. In this context, Italy took a special place, being the first European country after China to be affected by the pandemic, and many “emotional” commercials were released, always emphasising gratitude to those who fought on the front lines against the pandemic, such as doctors and service personnel, and celebrating national sentiment.

Emblematic of the extent to which the emotional sphere of the individual was emphasized was the commercial for the well-known Budweiser brand, created for the United States and devoted entirely to those who fought the pandemic. Based on a sports metaphor, the title of the commercial was *One Team* and celebrated the health care workers, the teachers, the soldiers, the athletes, the couriers, all those who faced the crisis. The commercial was actually a campaign in support of the American Red Cross (ARC): the company decided to redirect its planned investments in the sports market by donating $5 million to ARC and starting production of disinfectants in two of its plants, to be distributed to the blood donation centres of ARC.

With the same goal, Barilla, a major Italian brand in the food sector, created the commercial *All’Italia (To Italy)*, characterised by its strong patriotism. It was released on April 4, during the fourth week of the lockdown in Italy, and combined the most famous musical theme in the history of the company, imprinted in the memory of customers for almost 40 years, with images of the main Italian cities and the voice of Sophia Loren, one of the most iconic Italian actresses, as well as the hashtag #ItaliaCheResiste (#ItaliaFightingBack). This commercial represented a perfect combination of a brand with a symbol of Italy that was able to show its strength when the commercial was distributed abroad and became the perfect synthesis of the brand philosophy for foreign markets.

Another example came from Facebook, whose commercial appealed to the emotional sphere with images showing the desolation of the world’s major cities: it addressed loneliness and fear and then celebrated the solidarity and resilience of those fighting on the front lines and at home. The hidden message of the commercial was “we can’t get lost if we look for each other”. We could mention many more commercials dedicated to the common theme of building and strengthening confidence and hope, reinforcing a sense of community, and emphasising that “more than ever, health is in the hands of all of us”.

Based on these premises, the purpose of this paper is to investigate how the outbreak of the pandemic changed advertising messages by forcing the use of emotional content that was different from the content traditionally used, such as the product or brand name. This research objective was achieved indirectly by using a large corpus of tweets divided into three time periods—before, during, and after the lockdown—to measure consumer response to changes in advertising content during the most acute phase of the pandemic. The methods used are typical of text data mining, in which a text is considered as a finite part of an infinite stream of terms (Sebastiani 2002; Tandel et al. 2019). The order of occurrence of the terms is not relevant, only the frequency of occurrence of the terms is important to decode the semantics and sentiment contained in each tweet (Blei 2012).

The paper is organized as follows. Section 2 introduces the basic concepts, i.e. the main characteristics of advertising and brand awareness, and the essence of the Bag-of-Words model (BOW) as a basis for text data analysis. Section 3 describes the scraping of tweets and the preprocessing used to prepare the text corpus for subsequent analysis. Section 4 presents the results of a traditional term frequency analysis along with some extensions useful for a better understanding of the phenomenon under study. Section 5 documents the evolution of the semantics of tweets over time using an analysis over multiple periods that is equivalent to that possible with the Dynamic Topic Model (Blei and Lafferty 2006; Jähnichen et al. 2018) but uses a version of the mixture of Unigrams model (Nigam et al. 2000; Anderlucci and Viroli 2020) to account for the brevity of each tweet and the resulting sparseness in the BOW representation (which is inconsistent with the hypothesis that multiple topics are present in each tweet). Finally, in Sect. 6, we draw conclusions and suggest future research directions.

## Background

### Advertising and brand awareness

Companies invest large amounts of resources in advertising with the primary goal of making their products known and thus increasing their economic performance in terms of revenue. In a broader sense, advertising also helps build brand image and awareness. Advertising awareness can be described by whether consumers remember a brand’s advertising (Vaughan et al. 2016). Brand awareness refers to whether consumers remember or can recognize a brand, or simply whether consumers know about a brand (Keller 2013). It is also related to the identification with a particular company and affects the impact of advertising on consumers by influencing their reactions and perceptions (Bhattacharya and Sen 2003). The effects on the recipient of the advertisement include both affective and cognitive reactions. The first concerns preference for a particular product due to sentimental attachment, while the cognitive response concerns perception and memorization of the advertising content associated with a brand.

Cognitive response can be used to measure the effectiveness of an advertising campaign, as well as its ability to impress, be understood, and be remembered by its audience. Awareness measurements are often used in market research as a measure of brand performance and marketing effectiveness (Romaniuk et al. 2004). The most commonly used indicators related to brand and advertising awareness are: (i) recognition, i.e. the ability of consumers to identify a particular brand by its attributes over another; (ii) recall, i.e. how memorable an advertisement is to the audience; (iii) awareness, i.e. the percentage of target customers or accounts that show awareness (either with or without help). Advertising campaigns also aim to maximize “brand lift" increasing the perception and awareness of a product or brand over time in terms of recall and familiarity. The impact of an advertising campaign in terms of these attributes is typically measured directly, through consumer interviews and surveys, to understand and monitor the public’s awareness of a brand or product and to assess its commercial positioning and competitive strength in the relevant market.

In addition to television advertising, which is an important channel through which brands communicate with customers (Bruce et al. 2020), the media channel that has seen an advertising explosion in recent years is undoubtedly the Web (Fuxman et al. 2014). See, for example, Calder et al. (2009) for an examination of how consumer engagement on a website can increase the effectiveness of advertising. In recent years, there has also been an explosion of Web Analytics techniques aimed at gathering information valuable for understanding how the website works from the perspective of the user and the company (Xun 2015). All companies monitor their websites and the behaviour of users accessing the website based on a set of parameters automatically recorded by the provider or set by the company itself: the number of hits on each page of the website, the time spent on the website, the keywords entered through the search engines, the entry and exit pages. The same type of monitoring can be performed in real time on smartphones, tablets and social media platforms, opening up new interesting markets for advertising. The biggest change the Internet has brought, however, is represented by social media platforms, which have reshaped the way consumers create and distribute content through their ability to connect users with each other (Peng et al. 2018). Marketing communications through these platforms help companies reach new customers and increase demand for their products through user connections (Stephen and Toubia 2010; Schweidel and Moe 2014). On the Twitter platform, where the data for this analysis was collected, users retweet the content they read about an ad and build a bridge between the company that published the ad and new customers by sharing the ad with their followers (Gong et al. 2017).

These trends accelerated rapidly after the outbreak of the health emergency associated with the COVID-19 pandemic, which changed the everyday behaviour of citizens around the world and, consequently, the relationship between consumers and brands. On the one hand, consumers had to change their shopping habits, as they could no longer leave the house and shopped online whenever possible. On the other hand, brands had to rethink both their sales techniques and the way they advertised in order to appeal to audiences in difficult conditions. During the lockdown, messages of encouragement and solidarity were sent in all communication channels and even in advertising, as a kind of collective embrace that underlined the strength of the Italian people to defy adversity and emphasize those values that might have been taken for granted before, such as love, family, solidarity between people. Initially, there were only a few companies in Italy that designed advertising campaigns specifically for that time of crisis, and as the weeks went by, there were more and more. Some well-known companies in the field of telecommunications launched ad hoc commercials based on the message of shortening the distances to friends and relatives through video calls, phone calls, the Internet, the possibility of watching a movie or the favourite series TV, sharing emotions with family and friends. Advertisers directed messaging efforts toward risk communication strategies such as safe health and wellness practises (Deng et al. 2020).

The brands that have decided to change their approach were numerous and, to varying degrees, successful in creating empathy. For example, many car companies have moved in this direction: the FCA commercial titled *Anthem to the Home* or the Volkswagen brand, which used the hashtag #IoRestoInGarage (#Istayinthegarage). The Mazda video clarifies the shift in customer approach in the very first sentence: “This is not really a commercial, but a message of hope”. With a still image, a musical tapestry, poppy words and an absolutely minimalist style, the idea is to convey to customers that it is not about selling, but about being there, even if it still reminds them of the brand. Cosmetic companies have also released positive messages. A well-known makeup brand introduced the hashtags #torneràilsorriso (#smilewillcomeback) in its commercial with the song *Ritornerai*, showing a series of images of happy moments and memories of life before the emergency, such as visiting a city or going out with friends. It is therefore interesting to understand how consumers have responded to this change. In particular, we will study the patterns in Italian tweets in response to the aforementioned paradigm shift in advertising for some well-known brands in the food and consumer goods industries. This aspect makes the research design special and deviates from traditional approaches based on systematic reviews.

### The vector space model for text mining techniques

Using a corpus of tweets to analyze a particular research question requires that each tweet is represented in terms of a BOW model. In this simplified representation, both the grammar and the order of occurrence of words are irrelevant. It is only relevant whether a term occurs or not and how often it occurs in a text. Therefore, a number of preprocessing steps are required according to Natural Language Processing methods (NLP), detailed in the next section, to transform the raw text into a BOW representation (Manning and Schütze 1999; Manning et al. 2008). The final output is a vocabulary of terms *V* that forms the basis for further representations.

The simplest representation generates an indicator variable for each term in the vocabulary *V*, where 1 indicates the presence of the term in the tweet and 0 its absence (Manning et al. 2008; Wilbur and Kim 2009). This means that each document is represented as a binary vector $e_{1:\vert V \vert } = (e_1, \ldots , e_{\vert V \vert })$ with dimensionality $\vert V \vert$. The entire tweet corpus is therefore represented by a large sparse binary document term matrix (DTM) of dimension $\vert {{{\mathcal {D}}}} \vert \times \vert V \vert$, where ${{\mathcal {D}}}$ denotes the set of retrieved tweets.

For a more structured representation, we can introduce the frequency of occurrence of terms in a document by using the vector space model (VS), where documents are represented as vectors in ${\mathbb {R}}^{\vert V \vert }$ (Salton et al. 1975). The DTM can be weighted by the term frequency ${\textsf {TF}}_{td}$ of the word $t \in V$ in the tweet $d \in {\mathcal {D}}$ and by the term frequency-inverse document frequency:

1 $$\begin{aligned} {\textsf {TF-IDF}}_{td}={\textsf {TF}}_{td}\times {\textsf {IDF}}_t, \end{aligned}$$

which dampens the effect of local term-document counts. As usual, the inverse document count of the word *t* is defined as:

2 $$\begin{aligned} {\textsf {IDF}}_t = \log \left( \vert {{{\mathcal {D}}}} \vert / {\textsf {DF}}_t \right) , \end{aligned}$$

where ${\textsf {DF}}_t$ is the number of documents in ${{{\mathcal {D}}}}$ that contain the term $t \in \vert V \vert$ (document frequency). If the term *t* occurs in every document in the corpus, ${\textsf {IDF}}_t$ is 0. The fewer documents that contain the term *t*, the higher the value of ${\textsf {IDF}}_t$ (Nguyen 2014).

## Data preparation

The corpus of tweets used in this work was extracted using the Python library GetOldTweets3 (Mottl 2019), which overrides the official time limit of the standard API—due to which it is not possible to obtain tweets older than a week—by using a direct browser search for the given query and scraping the tweet informations. In advance, we created a list of search terms that included the names of the brands appearing in the commercials, the hashtags they launched, and more general search terms such as “TV commercial” or “coronavirus TV commercial”. The reference period for the analysis of the tweets was the period from 2020/01/01 to 2020/06/30. After extracting the raw data, the following fields were identified for each tweet: Date, ID, content of the tweet, the search terms matched during extraction.

A total of 21,017 tweets in Italian were identified, which were further processed using the NLTK Python toolkit (Bird et al. 2009) in the following steps:

- Removing tags (such as @, retaining the hashtag #) and URLs.
- Removing of punctuation marks.
- Removing strings consisting of sequences of the same letter.
- Removing numbers.
- Removing extra spaces.
- Converting to lowercase.
- Removing stop words.

Search terms found in the text of each tweet were also removed. After pre-processing, some tweets were removed because they were found to be empty, as they originally consisted of only a link, or they contained no text but only a hashtag. After this additional cleaning process, the total number of tweets in the corpus was reduced to 20,982. Finally, each tweet was tagged with a marker that referred to one of the following three time intervals:

- Phase before the lockdown: from 2020/01/01 to 2020/03/08.
- Phase during the lockdown: from 2020/03/98 to 2020/05/03 (on May 3, 2020, the so-called phase 2 begins).
- Phase after lockdown: from 2020/04/05 to 2020/06/30.

The cleaned tweets formed the basis of the corpus on which the study was conducted and were used for subsequent BOW analyses. The cleaned corpus was integrated with the following metadata: Date, ID, the time window defined above (before, during, and after), and the brand to which the tweet referred. To ensure reproducibility, both the cleaned corpus and its metadata are available from the authors upon request.

## Exploratory analysis

### Crude frequency analysis

All data analysis in this Section was performed using R 4.1.2 (R Core Team 2021), along with a set of libraries specifically designed for NLP and text mining, most of which are part of the Data Science infrastructure tidyverse (Silge and Robinson 2016; Wickham et al. 2019). To better understand whether and how attitudes toward television advertising have actually changed, a preliminary descriptive analysis of the number of tweets posted and words used during the analysed period was conducted. This analysis is based on the rule that the higher the media exposure, the more a brand is appreciated, and that collective awareness, regardless of reputation, is in itself an indicator that can also become a decision factor for consumers.

It is worth noting that a study by Kantar, one of the world’s leading market research and marketing consulting firms, found that Internet browsing increased by 70% in the later stages of the crisis, viewing of TV increased by 63%, and social media use increased by 61% over normal use (Kantar 2022). Only a very small proportion of consumers expected advertising to stop. Kantar estimated that a six-month absence from TV would result in a decline in brand awareness of about 39%, further delaying recovery after the emergency. Consumers also expected that brands that continued to advertise would have a positive impact on society: a reassuring tone and messages that would provide encouragement to overcome the difficult situation. In addition, 75% of respondents believed that brands should not use the crisis to advertise their products. This shift in the characteristics of advertising at the time of the pandemic is also well documented in our data. Looking at the frequency distribution of the number of tweets posted in each of the three time periods considered, we find the following:

- 3129 (14.91%) tweets in the pre-lockdown period (Pre).
- 11,423 (54.44%) tweets in the period during the lockdown (Dur).
- 6430 (30.65%) tweets in the period after the lockdown (Post).

These numbers are very significant from an awareness perspective: regardless of the content of the tweets and whether they expressed a positive or negative opinion, consumers tweeted 365% more during the lockdown than before the emergency.

**Fig. 1:**
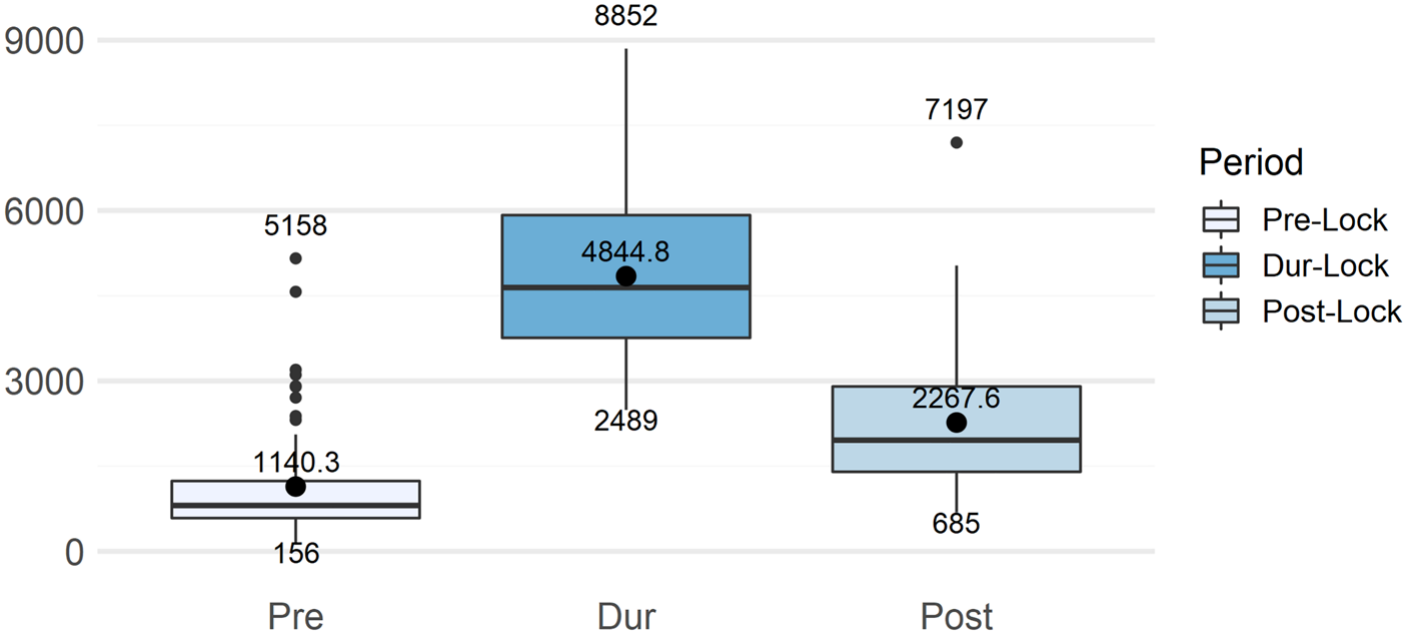
Boxplots of the average daily count of words used in tweets posted in each of the three time periods considered: before the lockdown (Pre-lock) from 2020/01/01 to 2020/03/08; during the lockdown (Dur-Lock) from 2020/03/98 to 2020/05/03; after the lockdown (Post-Lock) from 2020/04/05 to 2020/06/2020

This effect is also evident when we count the number of words tweeted daily. Figure 1 was created using whitespace tokenization of all tweets and counting the number of tweets used on each day. Finally, we plotted these data as boxplots by taking summary values for each of the three time periods considered. In the pre-emergency period, the average number of tweets per day in response to the brands’ commercials TV was 1140, while in the emergency period it increased to 4845. After the end of the most acute phase of the emergency, it decreased again, but without reaching the pre-crisis level (it averaged 2268 words).

**Fig. 2:**
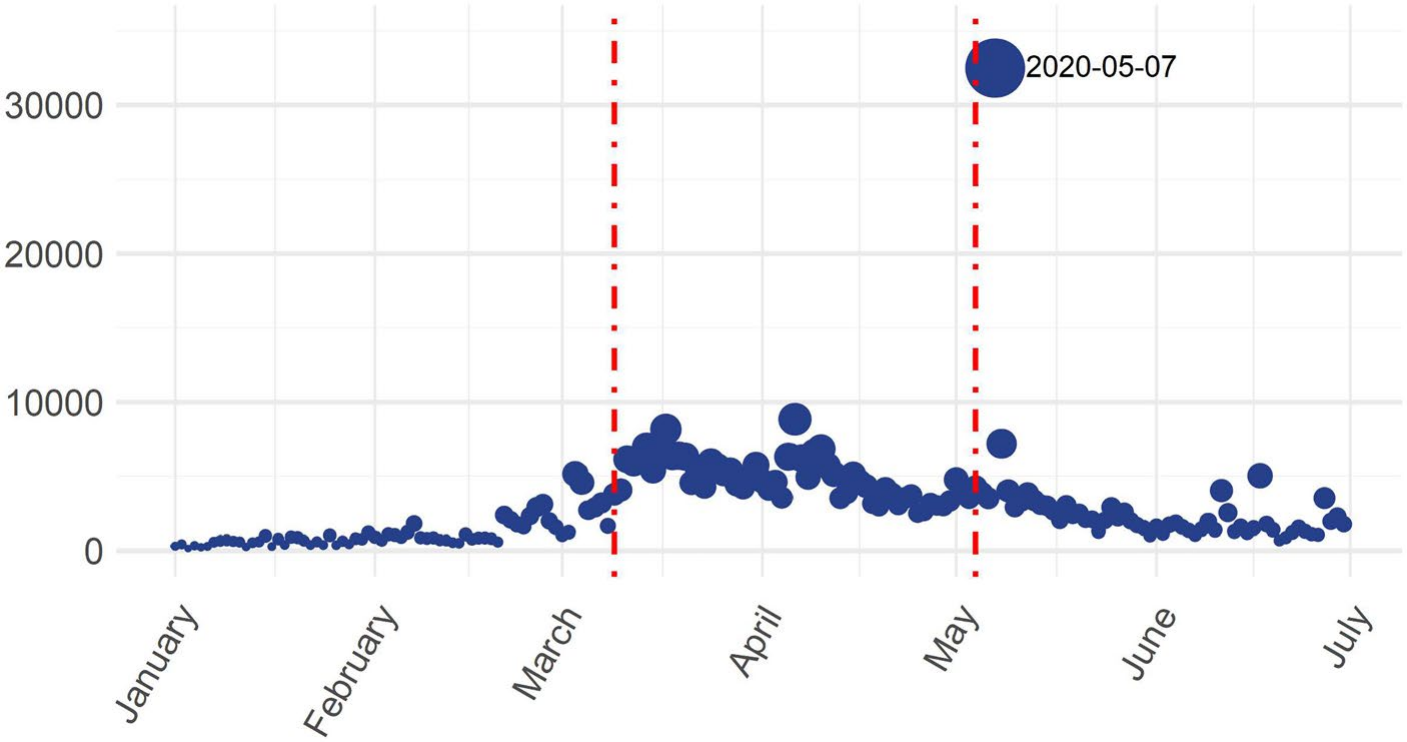
Daily historical series of the number of tweeted words in the time periods considered for the data analysis: before the lockdown (Pre-lock) from 2020/01/01 to 2020/03/08; during the lockdown (Dur-Lock) from 2020/03/98 to 2020/05/03; after the lockdown (Post-Lock) from 2020/04/05 to 2020/06/2020

In Fig. 1, the day 2020/05/07 was intentionally removed because it can be considered an outlier. The reason for this decision can be seen in the following graph (Fig. 2), which shows the historical series of the number of words tweeted daily. The outlier can be clearly seen frpm this figure. On May 7, the number of tweeted words exploded to an unprecedented level. To be exact, on May 7 we had exactly 32,496 words, of which 89.4% appeared in tweets that had the *Lavazza* brand as a reference tag (1060 tweets over 1199, with 29,046 words over 32,496). The hashtag #Lavazza reached the third position among the trends on Twitter in Italy that day and became a Trending Topic.

**Fig. 3:**
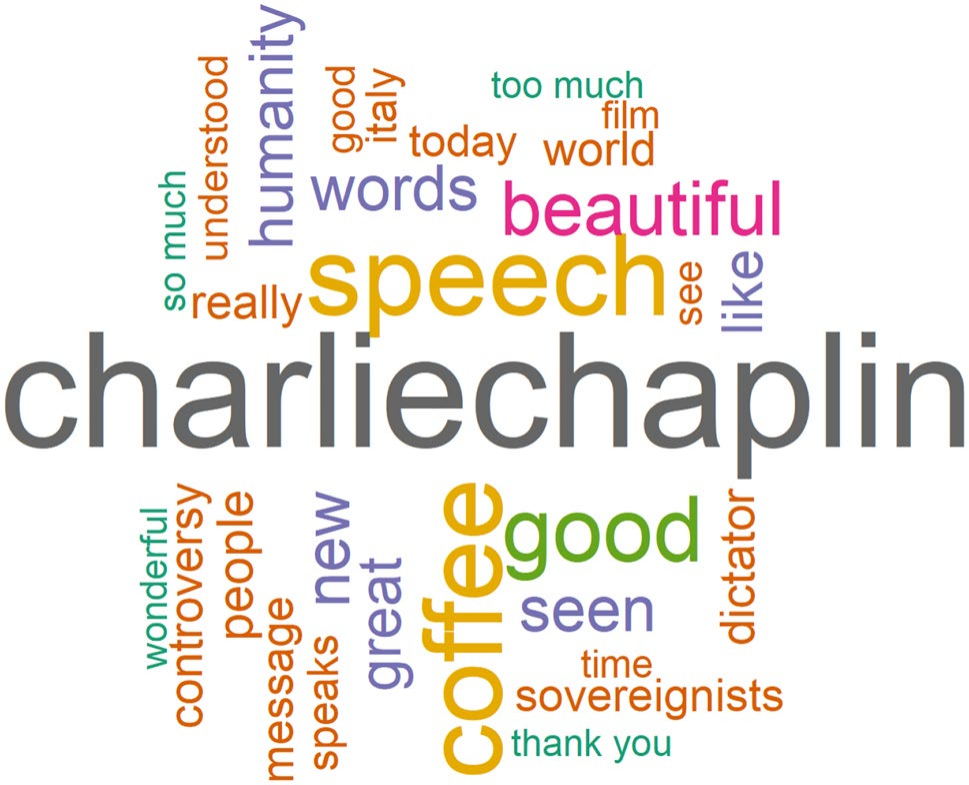
Wordcloud of the 30 most common terms tweeted in reference to the Lavazza spot on May 7, 2020

Lavazza’s commercial was released on May 7, 2020, under the title *Good Morning Humanity*. It was the starting point of a global communication campaign aimed at conveying a positive message through the words of Charlie Chaplin’s speech to humanity, presenting on the horizon a new world ready to welcome a rediscovered humanity. This aspect is well illustrated in the wordcloud in Fig. 3, which is based on the 30 most frequent words that appeared in tweets with Lavazza as the reference mark on May 7, 2020. As can be seen from the graph, the most frequently used tokens include positive adjectives such as beautiful, great or new, or words such as humanity and like and words with negative emotion polarity such as dictator and controversy. The most common word is charliechaplin, the absolute star of the commercial with his speech about humanity based on the famous movie *The Great Dictator*. The presence of terms with negative content is explained by the fact that this commercial has caused a wide debate among Twitter users. On the one hand, it was appreciated as a masterpiece that evokes positive feelings for humanity in crisis. On the other hand, it was strongly criticised because it was seen as a rhetorical display of good feelings and goodwill towards other people. However, it should be noted that Lavazza’s previous commercials were more humorous and focused on the goodness of the product, the opposite of this new commercial launched on May 7, 2020, which is based on the emotional message about the progress of humanity, revealing a very obvious paradigm shift in the structure of the message.

**Fig. 4:**
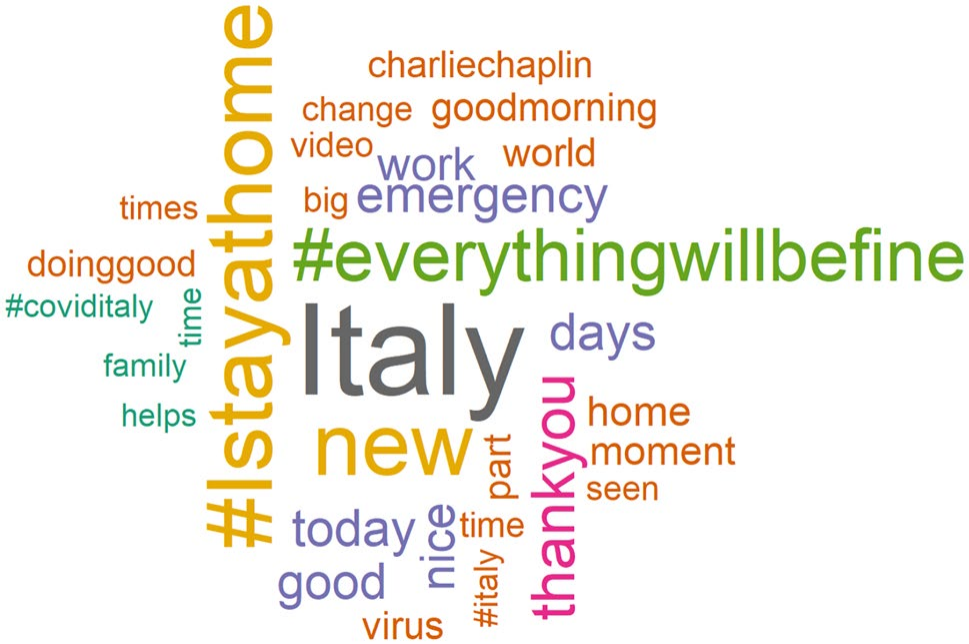
Wordcloud of the 30 most tweeted terms over the entire reference period from 2020/01/01 to 2020/06/30

The wordcloud of the 30 most frequent terms over the entire period considered for the analysis is shown in Fig. 4. It is immediately apparent that the most frequently used words in the comments on the TV commercials were #Istayathome and #everythingwillbefine, but also emergency, which perfectly fits that moment, and are justified by the massive use of such hashtags to comment on or end any written conversation. In particular, #Istayathome was launched by the social account of the Ministry of Health, which called for a massive spread. The call was welcomed by many brands, which in turn influenced consumers.

**Fig. 5:**
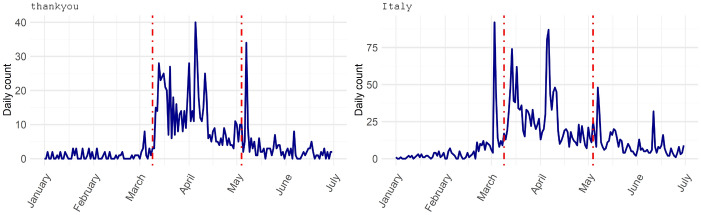
Daily time series of the number of tweeted terms thankyou and Italy in response to commercials from TV over the entire analysis period from 2020/01/01 to 2020/06/30

The words Italy, thankyou and new also play an interesting role. The latter (new) seems to refer to the ‘new world’ described in the speech of the great dictator from the Lavazza advertisement mentioned above. Moreover, the new advertising approach of the different brands seems to have been perceived as such and to have evoked a sense of appreciation and gratitude (thankyou) and a sense of belonging (Italy) among consumers. The evolution of thankyou and Italy over time confirms their particular use in the context of this contingent situation, as they accounted for no more than 1% of the relative daily frequency of tweeted words before the lockdown. These dynamic effects can be well documented by looking at the time series of daily occurrence of a particular term. Figure 5 shows the graphs for the terms thankyou and Italy as examples. In particular, thankyou peaks at 40 occurrences per day in the middle of the lockdown period and then drops dramatically to its usual average level before the lockdown. The word Italy shows a very similar trend.

### TF-IDF analysis

To further characterize the differences between the three selected subperiods, we created the TF-IDF weighted DTM, according to expression (1). The original matrix contained 39,610 terms with 204,894 non-zero entries and had an extreme sparsity of nearly 100%. To reduce sparsity, we removed all terms that occurred less frequently in the tweets, i.e. all terms $t \in V$ for which $\textsf {DF}_t < \vert {\mathcal {D}} \vert \times (1 - 0.99)$. The resulting matrix contained only 58 terms with 25,688 non-zero entries and an overall sparsity of 98%. The large reduction in the vocabulary of terms is not important at this stage, as we are only interested in the words that are at the top of the list in terms of their frequency of occurrence adjusted for inverse document frequency.

**Fig. 6:**
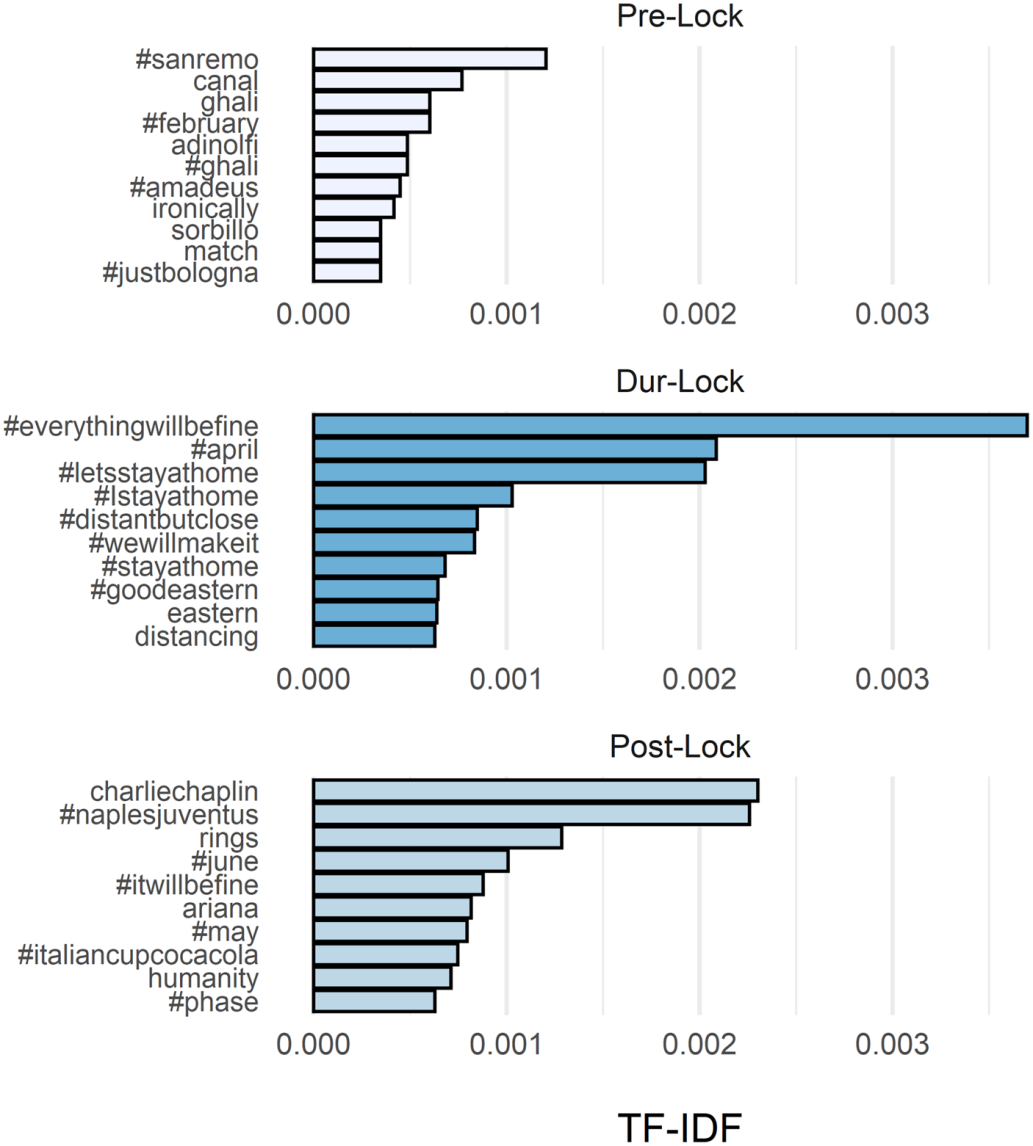
Barplots of the top-10 most frequents words, adjusted for the inverse-document frequency for each of the three time periods considered: before the lockdown (Pre-lock) from 2020/01/01 to 2020/03/08; during the lockdown (Dur-Lock) from 2020/03/98 to 2020/05/03; after the lockdown (Post-Lock) from 2020/04/05 to 2020/06/2020

The top 10 terms for each of the three time periods considered are shown in the bar graphs in Fig. 6. During the lockdown, as noted before, the most common words were hashtags of encouragement (#everythingwillbefine, #Istayathome, #wewillmakeit, #distantbutclose), which do not normally appear frequently in comments on television commercials. Not all terms that occurred most frequently during the lockdown were also frequently used after the lockdown. This initial rough dynamic analysis seems to indicate that the change in attitude toward advertising that occurred during the lockdown appears to be temporary. In fact, after the lockdown, we can observe a significant decrease in the frequency of terms that had characterized the lockdown period, and the reappearance of terms related to sports events (such as #naplesjuventus or #italiancupcocacola). It is very likely that in this phase commercials were again considered not as a moment of aggregation or expression of values of national solidarity, but as elements of disruption of television programs.

## Dynamic analysis of topic evolution

The goal of this section is to perform a dynamic analysis to examine the evolution of the probability distribution over the vocabulary of terms and actually document whether or not the paradigm shift in attitudes toward advertising was temporary. In the field of text data analysis, there are a number of models that are useful for analyzing the evolution of thematic content over time in large document collections. These models are based on latent topic models, the precursor of which is the Latent Dirichlet Allocation (LDA) model, in which a document is generated by sampling a mixture of these topics and then selecting words from that mixture (Blei et al. 2003; Blei 2012). Dynamic versions extend the static version of this model by adding a hierarchical level to the model in which the parameters that determine the distribution of topics change over time (Blei and Lafferty 2006; Hida et al. 2018).

Despite their excellent results on medium or large texts—typically emails and news that are on the order of $> 50$ words per document—both static and dynamic latent topic models perform poorly on short texts such as tweets because the assumption that a text is a mixture of topics cannot be applied. Therefore, our multi-period topic analysis will be based on a variation of the mixture of Unigrams model, which has been shown to be particularly efficient at unsupervised classification of short text data represented as a large document-term matrix with high sparsity (Anderlucci and Viroli 2020).

### Hierarchical Dirichlet-Multinomial mixtures

The model we used for the dynamic analysis is the following hierarchical Dirichlet-Multinomial multinomial mixture, introduced and studied in detail in the companion paper by Bilancia et al. (2022):

3 $$\begin{aligned} y_i^{(t)} \vert \beta ^{(t)}, z_i^{(t)}&{\mathop {\sim }\limits ^{\mathsf {ind.}}} \mathsf {Multinomial}_{p}(\beta _j^{(t)}),\quad i=1,2,\ldots ,n^{(t)} , \end{aligned}$$

4 $$\begin{aligned} z_i^{(t)} \vert \lambda ^{(t)}&{\mathop {\sim }\limits ^{\mathsf {ind.}}} \mathsf {Multinouilli}_{k}(\lambda ^{(t)}),\quad i=1,2,\ldots ,n^{(t)} ,\end{aligned}$$

5 $$\begin{aligned} \beta _j^{(t)} \vert \theta ^{(t)}&{\mathop {\sim }\limits ^{\mathsf {ind.}}} \mathsf {Dirichlet}_p({\mathbbm {1}}_p\theta ^{(t)}), \quad j = 1,2,\ldots ,k, \end{aligned}$$

6 $$\begin{aligned} \lambda ^{(t)}\vert \alpha ^{(t)}&\;\sim \mathsf {Dirichlet}_k({\mathbbm {1}}_k\alpha ^{(t)}), \end{aligned}$$

where $t = 1,2,\ldots , T$ indicates the time period. The *p*-dimensional vector, where $p = \vert V \vert$:

$$\begin{aligned} y_i^{(t)} = \big (y_{i1}^{(t)}, y_{i2}^{(t)}, \ldots , y_{ip}^{(t)}\big )^{\top }, \end{aligned}$$

contains the counts of terms in *V* for the *i*-th tweet of the corpus used for the time period *t*. The hyperparameters $\alpha ^{(t)},\theta ^{(t)} > 0$ are strictly positive real numbers and ${\mathbbm {1}}$ denotes a vector of all ones.

Each $\beta _j^{(t)} \in {\mathbb {R}}^p$ is a vector of Multinomial parameters representing a topic, i.e., a probability distribution of the vocabulary of terms *V*. Different tweets may have different thematic content, i.e. terms or hashtags that appear in one tweet may be of little importance or not appear at all in another tweet. Since we do not know to which class each document belongs, the index of the row vector $\beta _j^{(t)}$ corresponds to the index of the single element in the latent indicator vector $z_i^{(t)} \in {\mathbb {R}}^k$ such that $z_{ij}^{(t)}=1$ (all other elements are zero), while $\lambda ^{(t)} \in {\mathbb {R}}^k$ denotes the mixing weights (Robert 2007). Since only one element of the vector $z_i^{(t)}$ is non-zero, the Multinouilli distribution is a Multinomial distribution for a single trial.

The hyperparameters $\theta ^{(t)}$ and $\alpha ^{(t)}$ are assumed to be fixed and empirically determined. The exchangeable Dirichlet prior over the Multinomial parameters implicitly gives the data more weight in updating the posterior distribution of each $\beta _j^{(t)}$ with an appropriate choice of the concentration parameter $\theta ^{(t)}$. Similar considerations apply to the mixture weights $\lambda ^{(t)}$. While Bayesian posterior inference is the natural framework for parameter estimation of the model proposed above, its applicability is complicated by the fact that the marginal distribution of the data cannot be written in closed form due to the high-dimensional integrations that enter the calculations. Therefore, we must resort to appropriate numerical methods for Bayesian estimation of the model parameters.

### Variational estimation

The approach we used for inference is that of variational inference (Blei et al. 2017), in which the joint posterior distribution is approximated by a probability distribution where the model parameters are assumed to be independent a posteriori. For simplicity, we suppress the explicit dependence on the time index *t* in this section. Moreover, the Multinomial parameters $\beta _j$ are stacked by rows in the matrix $\beta \in {\mathbb {R}}^{k\times p}$, where $\beta = \{ \beta _{j\ell } \}$ for $j=1,2,\ldots ,k$ and $\ell =1,2,\ldots ,p$. The vectors $z,\lambda$ and *y* collect all the remaining parameters and the sample observations.

In the synthesis, the starting point is the approximation of the posterior distribution $p(\beta ,z,\lambda \vert y, \theta ,\alpha )$ by a variational distribution $q(\beta ,z,\lambda \vert \nu )$, which itself depends on the variational parameters $\nu$. Our optimization problem is:

7 $$\begin{aligned} \nu ^\star =\mathop {{{\mathrm {argmin}}}}\limits _\nu \mathsf { KL }\left( q(\beta , z, \lambda \vert \nu ) ||p(\beta ,z,\lambda \vert y, \theta ,\alpha ) \right) , \end{aligned}$$

where the variational objective is the reverse Kullback-Leibler (KL) divergence between the posterior distribution and the variational distribution (Murphy 2013). The solution of (7) provides the best possible approximation to the intractable posterior distribution. The search for the optimal approximating distribution can be greatly simplified if we define the Evidence Lower Bound (ELBO) as follows:

8 $$\begin{aligned} \mathsf {ELBO}(q) = {\mathsf {E}}_q\left[ \log p(y,z, \beta , \lambda \vert \theta , \alpha ) \right] - {\mathsf {E}}_q\left[ \log q(\beta ,z,\lambda \vert \nu ) \right] , \end{aligned}$$

because minimizing the KL divergence with respect to the variational parameters is equivalent to maximizing the ELBO (which is a sum of linear terms when calculated in explicit form) with respect to the variational parameters (Zhang et al. 2019; Tran et al. 2021).

We specify the variational distribution $q(\beta ,z,\lambda \vert \nu )$ using a classical mean-field approximation with independent components (Blei et al. 2017):

$$\begin{aligned} q(\beta ,z,\lambda \vert \nu ) =\prod _{j=1}^k q(\beta _j \vert \phi _j) \times \prod _{i=1}^n q(z_i \vert \gamma _i) \times q(\lambda \vert \eta ), \end{aligned}$$

where $$\phi _j \in {\mathbb {R}}^p$$, $$\gamma _i,\eta \in {\mathbb {R}}^k$$, and:

9 $$\begin{aligned} \beta _j \vert \phi _j&{\mathop {\sim }\limits ^{\mathsf {ind.}}} \mathsf {Dirichlet}_p(\phi _j),\quad j=1,2,\ldots ,k \end{aligned}$$

10 $$\begin{aligned} z_i \vert \gamma _i&{\mathop {\sim }\limits ^{\mathsf {ind.}}} \mathsf {Multinouilli}_k(\gamma _i),\quad i=1,2,\ldots ,n \end{aligned}$$

11 $$\begin{aligned} \lambda \vert \eta&\;\sim \mathsf {Dirichlet}_k(\eta ). \end{aligned}$$

An explicit expression of the ELBO for this choice of the variational distribution can be found in Bilancia et al. (2022). In addition, an iterative coordinate ascent algorithm is described to find the parameter values that optimize the ELBO and correspond to the best possible approximation to the joint posterior distribution of the model parameters. Taking advantage of the fact that the variational distribution of each $\beta _j$ is Dirichlet, approximate posterior estimates of the probability distributions over the vocabulary of terms *V* can be computed using the following expression:

12 $$\begin{aligned} \beta _{j\ell }^\star =\frac{\phi _{j\ell }^\star }{\sum _{\ell =1}^p \phi _{j\ell }^\star }, \end{aligned}$$

and can be compared over time, as shown in the next subsection.

### Data preparation and results

The sequence of time-indexed models, which have the hierarchical structure presented in Sect. 5.1, represents a seemingly unrelated set of models, since no mechanism is provided for dynamically modeling latent parameters over time. This possibility would be essentially redundant for our purposes, since the goal is to compare the occurrence probabilities of terms in a vocabulary *V* common to all periods without unnecessarily overparameterizing the model.

For this purpose, we divided the observation period from 2020/01/01 to 2020/06/30 into $T=12$ subperiods of about fifteen days, as shown in Table 1. The frequency distribution of the number of tweets belonging to each period clearly shows that social media usage increased exponentially during the most acute phases of the lockdown, from 297 (1.42%) tweets in period 1 to 3757 (17.91%) in period 6. This last period corresponds to the most acute phase of the lockdown and emergency, when the stay-at-home mandate was extended to the entire national territory and all non-essential productive activities were stopped. The common vocabulary *V* was constructed based on the global document term matrix for all 20,982 tweets, taking only $t \in V_{\text {full}}$ terms for which $\textsf {DF}_t < \vert {\mathcal {D}} \vert \times (1-0.997)$, for a total of $p = 449$ terms, with an overall matrix sparsity of 99%. This choice was empirically calibrated and was based on a tradeoff between the computational complexity of model estimation and the informativeness of the term vocabulary *V*.

We applied our model with $k = 2$ and used the default values $\alpha ^{(t)} = 1$ and $\theta ^{(t)} = 5 / k$ for all *t*. This minimal choice of the number of topics is the simplest possible, avoids introducing unidentified components into the model, and assumes the existence of a main topic versus a background topic that collects the occurrence of terms that do not capture the dominant semantics of the time period we are considering. In this way, we can also filter out noise without violating the assumption that a short text such as a tweet cannot be considered as a mixture of latent topics, since each tweet is hard-clustered to a single topic. The choice of hyperparameters is neutral with respect to $\alpha ^{(t)}$ and uninformative with respect to $\theta ^{(t)}$. In the absence of additional information that would justify a different choice, they appear reasonable, leaving most of the responsibility for updating the posteriori distribution to the data.

**Table 1:** The $T=12$ subperiods considered for the analysis, with the corresponding absolute ($n_t$) and percentage (%) frequency distribution of the number of tweets in each period

*t*	Period	$n^{(t)} (\%)$	$\lambda _{1}^{(t)\star } \times 100$	$\lambda _{2}^{(t)\star } \times 100$
1	(from 2020-01-01 to 2020-01-15)	297 (1.42)	0.48	99.52
2	(from 2020-01-16 to 2020-01-31)	416 (1.98)	92.09	7.91
3	(from 2020-02-01 to 2020-02-14)	543 (2.59)	5.53	94.47
4	(from 2020-02-15 to 2020-02-28)	982 (4.68)	95.88	4.12
5	(from 2020-03-01 to 2020-03-15)	2525 (12.03)	73.64	26.36
6	(from 2020-03-16 to 2020-03-31)	3757 (17.91)	72.60	27.40
7	(from 2020-04-01 to 2020-04-15)	3419 (16.29)	42.24	57.76
8	(from 2020-04-16 to 2020-04-30)	2064 (9.84)	70.73	29.27
9	(from 2020-05-01 to 2020-05-15)	3514 (16.75)	73.61	26.39
10	(from 2020-05-16 to 2020-05-31)	1375 (6.55)	96.75	3.25
11	(from 2020-06-01 to 2020-06-15)	995 (4.74)	95.31	4.69
12	(from 2020-06-16 to 2020-06-30)	1095 (5.22)	5.18	94.82

The last two columns provide the estimates of the mixture weights ($k=2$) for each period

**Fig. 7:**
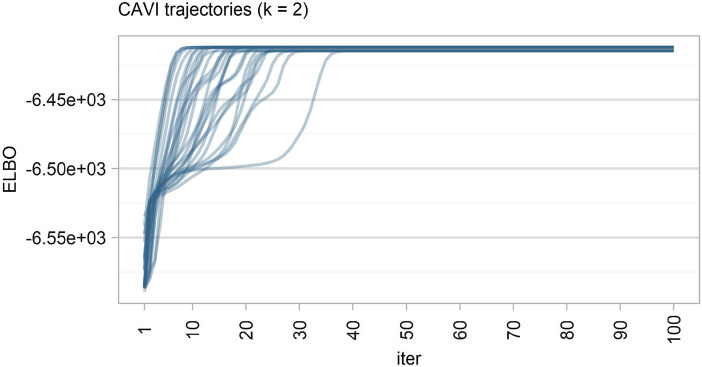
Some trajectories of the variational algorithm applied to the tweet corpus of period 2 (from 2020-01-16 to 2020-01-31)

We applied the algorithm for a total of 100 runs, starting from randomly chosen initial values. In Fig. 7 we show a subset of the trajectories of the coordinate-ascent variational inference (CAVI) algorithm. It is obvious that each run reaches a different stationary point, since we have many different local maxima in the ELBO. Better local optima lead to a variational approximation closer to the exact posterior (Plummer et al. 2020). Table 1 also shows the estimates of the mixture weights: As can be seen, the main component is often interchanged between $\lambda _1^\star$ and $\lambda _2^\star$. This phenomenon is a manifestation of the well-known label switching problem (Diebolt and Robert 1994). Since the likelihood of the model is a sum of terms invariant to each of the *k*! possible permutations of the summands, if we assume an exchangeable prior over the parameters, this invariance is inherited from the posterior distribution, which has *k*! symmetric modal regions corresponding to all possible permutations of the parameter labels. However, variational inference, like any optimization algorithm, depends on the initial conditions and focuses on one of the possible *k*! modes of the posterior surface depending on these conditions. Of course, this is not a drawback, because we know that a single mode contains all the information for exploring latent groups and estimating parameters, and the order of labelling is not relevant (Blei et al. 2017).

**Fig. 8:**
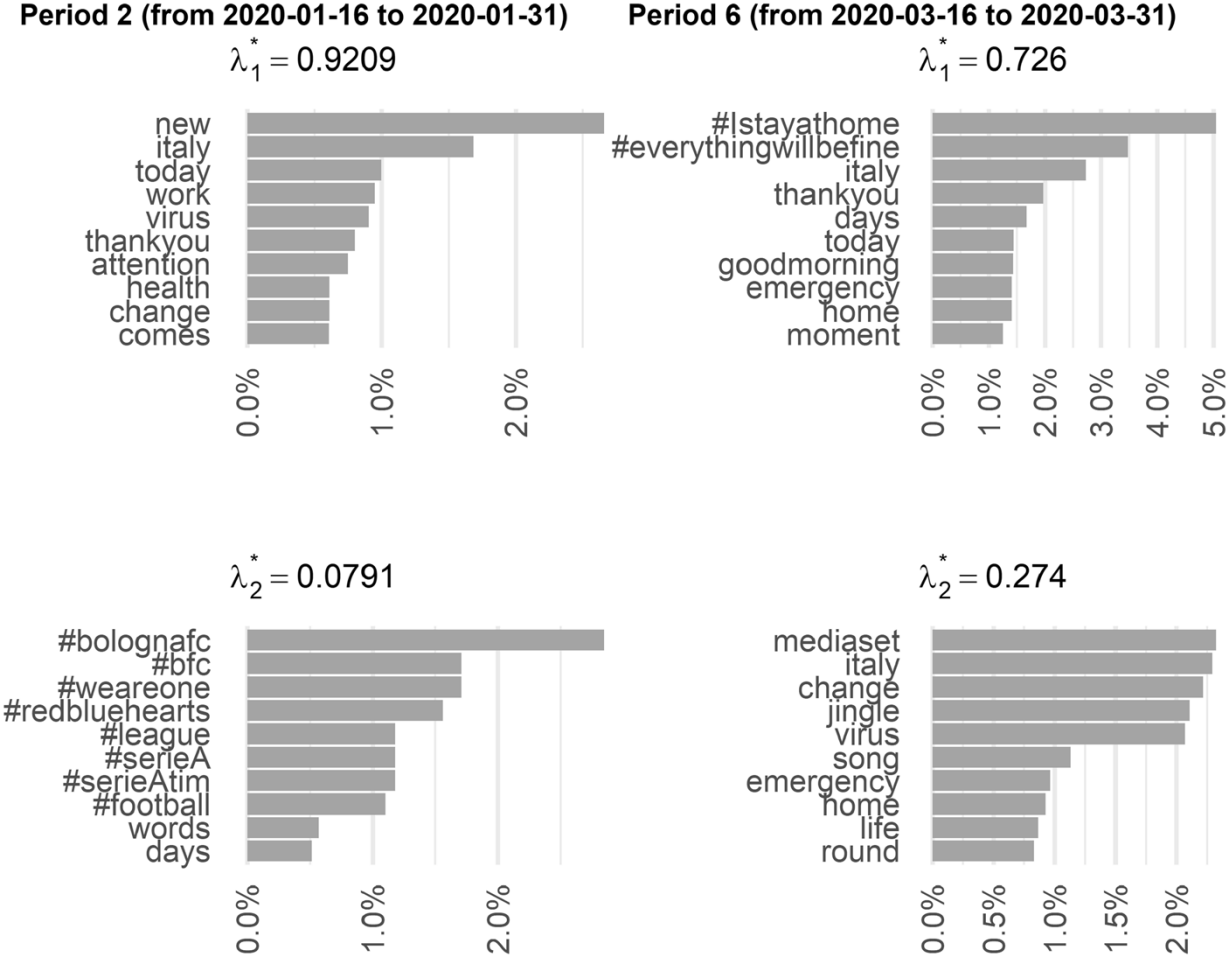
Top-10 terms of the estimated $\beta _j$ distribution for period 2 (from 2020-01-16 to 2020-01-31) and period 6 (from 2020-06-16 to 2020-06-30)

After this necessary technical introduction, we can examine the results by comparing the estimates of the probability distributions $\beta _j^{(t)}$. In Fig. 8, we compared period 2 (from 2020-01-16 to 2020-01-31) with period 6 (from 2020-03-16 to 2020-03-31). In period 2, we have a dominant component (the relative mixing weight is about 92%) in which the top terms are consistent with the fact that the first news of virus circulation and health emergency in China spread at this time (the first two confirmed cases of SARS-CoV-2 infection in our country involved two Chinese tourists and are dated January 31, 2020). The secondary component shows that the top-10 terms mainly concern sports events such as the soccer championship. In period 6, the mandate to stay at home obviously led to much greater use of social media and TV. The top terms in component 1 are clearly related to the emotional dimension of gratitude and the desire for the emergency to end, which peaks in these days and then begins a slow descent. Note, on the other hand, that in the background component the references to sports events have completely disappeared (in Italy the soccer championship was canceled on March 9, 2020). On the contrary, it is worth noting the appearance of terms such as #mediaset (one of the main commercial TV operators) and #jingle, proving that TV commercials played an important role in conveying the widespread emotions during this period of the pandemic. These data, matched with raw frequency analyses, confirm that the role of advertising on TV did indeed change during the lockdown and was perceived as a vehicle for solidarity and positive emotions to bridge the worst period of the pandemic in our country.

**Fig. 9:**
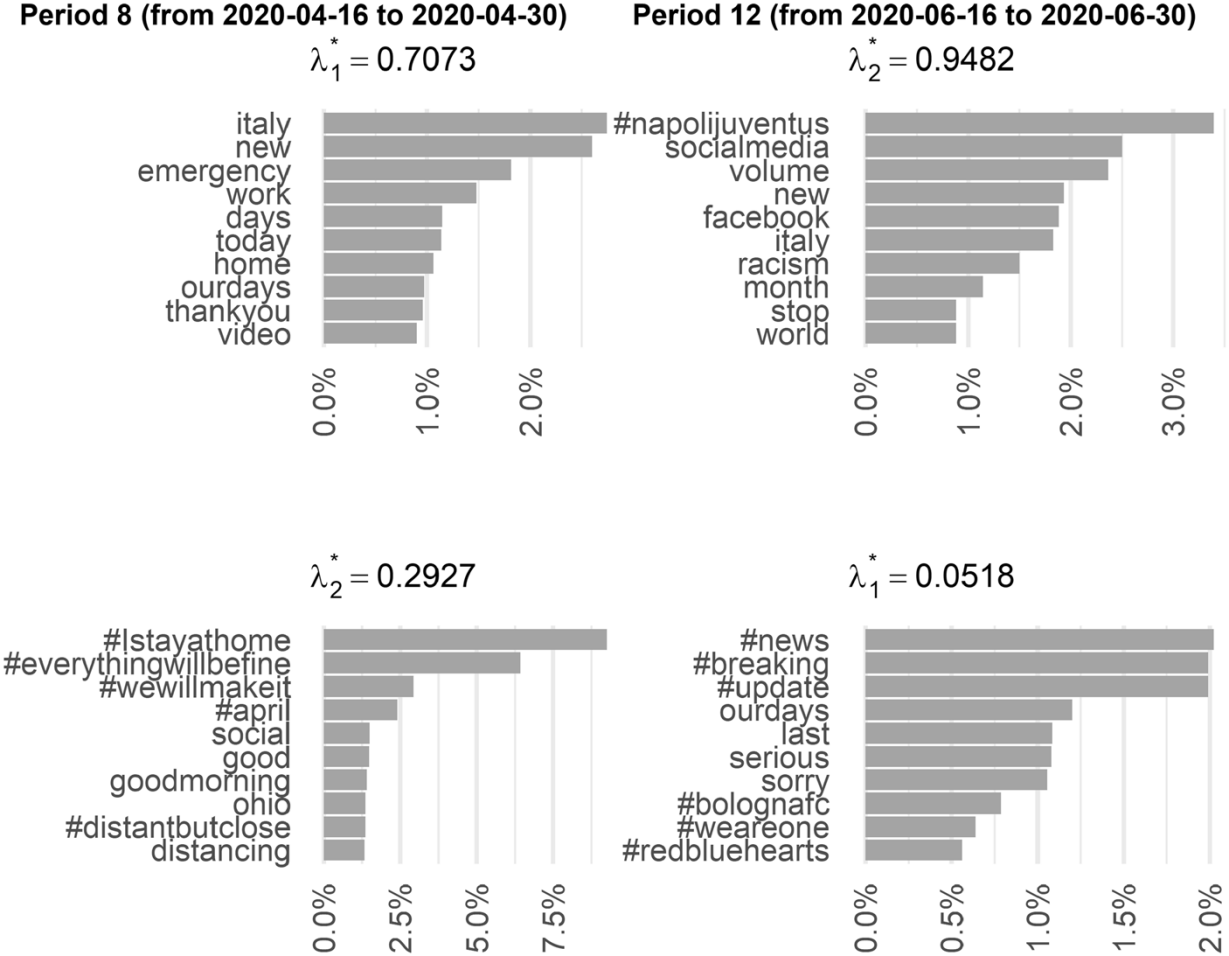
Top-10 terms of the estimated $\beta _j$ distribution for period 8 (from 2020-04-16 to 2020-04-30) and period 12 (from 2020-03-16 to 2020-03-31)

However, this phenomenon is only temporary. In Fig. 9 we compare period 8 (from 2020-04-16 to 2020-04-30) with period 12 (from 2020-06-16 to 2020-06-30). In period 8, the lockdown was coming to an end. Beginning on May 3, 2020, there was an initial gradual easing of the lockdown such as the reopening of public parks, the resumption of food deliveries, and the resumption of various manufacturing activities. Only movements motivated by proven necessities were allowed (work, emergencies, and meetings with family), subject to the prohibition of gatherings with a minimum interpersonal distance of at least 1 meter. Looking at the distribution of the top-10 terms in both the main and background components, terms related to emergency, feelings of gratitude, and national cohesion continue to dominate. However, the reference to the media becomes weaker, with the term #social appearing only in the background component. The situation becomes even more evident in period 12, considering that since June 11, 2020, containment measures have been further relaxed and gambling and betting offices, theaters and cinemas, cultural and social centers were reopened (and since June 3, mobility between regions was fully possible again). This situation is reflected in the top-10 terms: although direct references to social media or Facebok are present, the most important term refers to a sports event, while those related to the dimension of emotion and gratitude have largely disappeared.

**Fig. 10:**
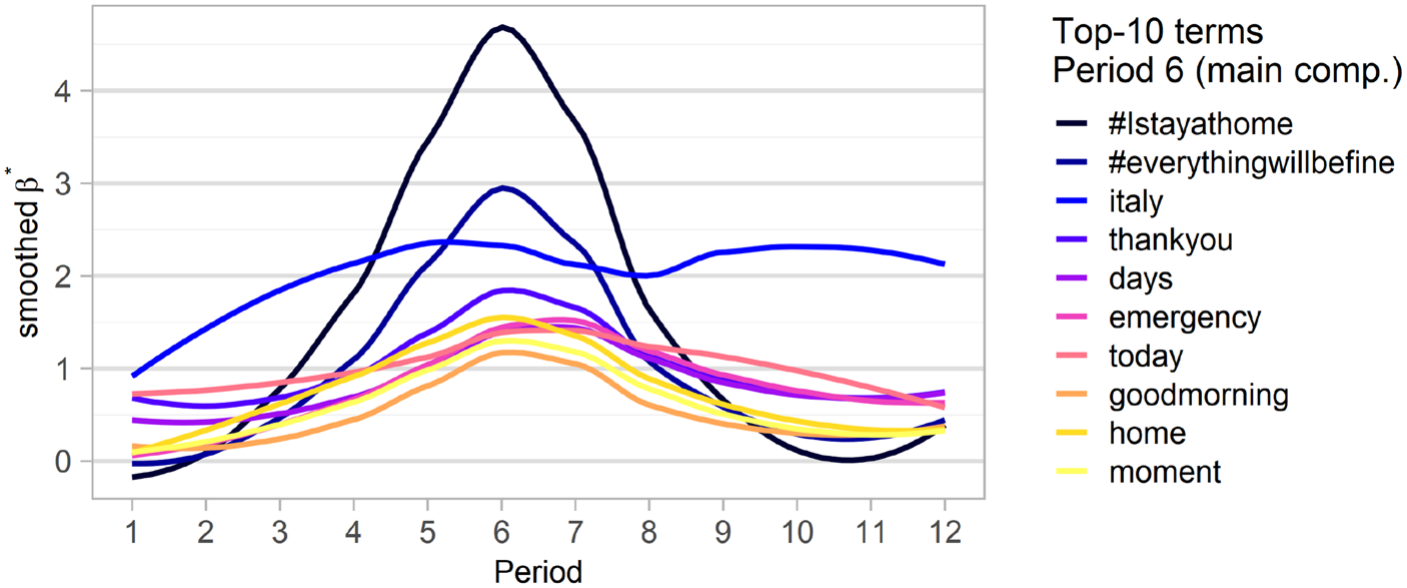
Estimated probabilities of occurrence (multiplied by 100) of the top-10 terms of period 6, for each of the $T=12$ terms considered. Values were smoothed using a loess smoother

To further confirm this analysis, we have presented in Fig. 10 the estimates of the top-10 terms of period 6 for each of the 12 periods considered (using the main component). These estimates have been smoothed with the loess smoother, which is why they may be just below zero at the extremes of the time curve. It is quite clear that both the hashtags associated with emergency response and those associated with feelings of gratitude and national cohesion peak during the two main periods of lockdown (periods 6 and 7, from mid-March to mid-April 2020) and then decline rapidly as the summer period begins and containment measures are relaxed (with the notable exception of Italy, whose probability of occurrence remains roughly stable throughout the period considered). Thus, although the link between some frequently occurring terms and the new dimension of advertising campaigns launched during the most acute phase of the emergency is undeniable, it is equally obvious that this effect is temporary and does not correspond to a permanent paradigm shift in advertising.

**Fig. 11:**
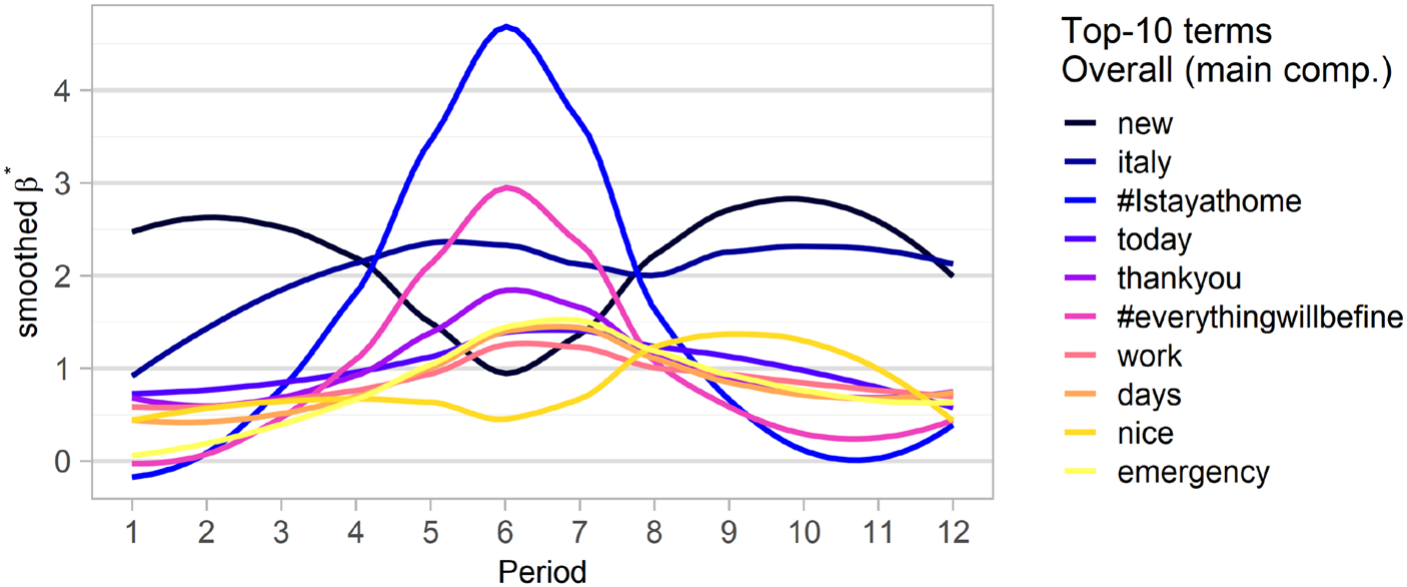
Estimated probabilities of occurrence (multiplied by 100) of the top-10 terms with the highest average probability of occurrence, for each of the $T=12$ terms considered. Values were smoothed using a loess smoother

To provide additional evidence, we plotted a similar graph in Fig. 11, but we used the top-10 terms with the highest average probability of occurrence (calculated as the average of the estimated probabilities of occurrence in each period) for the entire period considered. Note that most of the terms are the same as in the previous figure, or the temporals trends are qualitatively similar, confirming that the peak occurrence of the top terms is a transient phenomenon characterized by a bell-shaped temporal curve (in most cases). A noteworthy aspect is that the top term in this case is new, which has an unusual temporal trend with two peaks in the first and last parts of the curve. In the first phase, the spike in occurrence can certainly be related to the fact that the virus was initially named as the ‘new 2019 coronavirus’ (2019-nCoV) and then renamed to its current name SARS-Cov-2 in February 2020. On the contrary, as we have already noted, the second spike could apparently be related to the new world described by the Great Dictator in the influential Lavazza advertisement, or even to the new phase of the pandemic beginning on May 3, 2020, at the end of the most severe phase of the emergency.

## Discussion and conclusions

Understanding the socioeconomic impact of social media and new technologies on the way people think is of interest not only to researchers, but also to governments and business managers. With the advent of Big Data, a growing number of academic papers propose the use of text mining techniques to analyse Web data, especially unstructured text such as comments and ratings (Georgieva-Trifonova and Dechev 2021). However, real-time emotion recognition is an evolving area of research, particularly with respect to the theoretical underpinnings of artificial intelligence-based techniques (Lee and Park 2018). Social media has changed the traditional way of communication between brands and consumers. The latter have the power to exert a positive and negative influence on the brand, for example, through their reviews. Therefore, it is important for companies to manage social media communication by building brand awareness and a positive image. An additional finding of this study was evidence that companies can use social networks to monitor brand perceptions and build brand awareness and image using appropriate text mining techniques. Internet advertising is preferable to traditional media such as newspapers, TV and radio, and marketing is currently paying the most attention to interaction with social channels (Awad Alhaddad 2015).

The recent COVID-19 pandemic has led to new patterns of behaviour that differ from previous ones in terms of responses and emotions to external stimuli. Indeed, the way a society perceives the stimuli emanating from its environment is an input that comes directly from advertising. The main results of this work confirm that the major brands changed their communication strategy and based their commercials TV on the principles of solidarity and fraternity, in order to increase trust and hope in Italian families during the pandemic response. The formats of their commercials were inevitably changed and triggered reactions from customers, as measured by the increasing number of tweets and their content. Companies hoped to strengthen their brand image by spreading empathetic messages to customers who needed trust at a time of extreme emotional fragility. The article aims to measure customer responses expressed on the Twitter social network.

These changes have been documented using text data mining techniques that arise from the need to automate the understanding of unstructured textual data found not only in books and newspapers, but also in posters, blogs, customer reviews on the Internet, and posts on social media. Our proposal addresses an ad hoc corpus of unstructured data and transforms it to perform text mining analysis and identify patterns and trends. After a careful phase of extraction and preprocessing of tweets using NLP algorithms, a dataset of 20,982 tweets was created and analysed, considering three different time periods: before the lockdown, during the lockdown, and after the lockdown. Frequency analysis by the TF-IDF algorithm confirmed the expected extraordinary increase in activity on the Twitter social network during the lockdown, but most importantly a completely different use of the terms Italy, thankyou, and new, which were among the most frequently used in tweets, along with the hashtags #Istayathome and #everythingwillbefine. Thus, the novelty of the brand’s advertising approach seems to have primarily created a sense of gratitude and belonging that was not present before, as TV commercials were usually perceived as disruptive elements.

It should be noted, however, that the effects we documented proved to be essentially transitory when we used a dynamic model to study the evolution of the probability of occurrence of the most frequent terms as a function of time. Indeed, from the end of the lockdown onward, there is a clear decrease in the prevalence of terms with emotional content or praising national solidarity (which can clearly be associated with some of the most famous commercials from this period). It is not clear, and this aspect will be the subject of future studies, whether there are enduring aspects in the current paradigm of TV commercials and in consumer response to them that have definitely changed as a result of the health emergency and the lockdown. A final, but no less important aspect that is also currently under investigation and that we will address in future papers, is the investigation of the actual casual relationships that exist between the nonpharmacological interventions (NPIs, such as the lockdown) implemented in 2020 against COVID-19 and the variation in the number of words tweeted and/or their sentiment. From this point, classic quasi-experimental approaches such as regression discontinuity design or newer dynamic time series-based approaches, such as the one described in Brodersen et al. (2015), can be used to obtain interesting results.

## References

[CR1] Anderlucci L, Viroli C (2020). Mixtures of Dirichlet-multinomial distributions for supervised and unsupervised classification of short text data. Adv. Data Anal. Classif..

[CR2] Awad Alhaddad A (2015). The effect of advertising awareness on brand equity in social media. Int. J. e-Educ. e-Bus. e-Manage. e-Learn..

[CR3] Bhattacharya C, Sen S (2003). Consumer-company identification: a framework for understanding consumers’ relationships with companies. J. Mark..

[CR4] Bilancia, M., Di Nanni, M., Manca, F., et al.: Variational Bayes estimation of hierarchical Dirichlet-multinomial mixtures for text clustering (2022) (submitted)

[CR5] Bird, S., Klein, E., Loper, E.: Natural Language Processing with Python (2009). http://nltk.org/book/

[CR6] Blei DM (2012). Probabilistic topic models. Commun. ACM.

[CR7] Blei, D.M., Lafferty, J.D.: Dynamic topic models. In: Proceedings of the 23rd International Conference on Machine Learning—ICML ’06. pp. 113–120. ACM Press, New York, New York, USA (2006). 10.1145/1143844.1143859

[CR8] Blei DM, Ng AY, Jordan MI (2003). Latent Dirichlet allocation Michael I. Jordan. J. Mach. Learn. Res..

[CR9] Blei DM, Kucukelbir A, McAuliffe JD (2017). Variational inference: a review for statisticians. J. Am. Stat. Assoc..

[CR10] Bruce NI, Becker M, Reinartz W (2020). Communicating brands in television advertising. J. Mark. Res..

[CR11] Brodersen KH, Gallusser F, Koehler J, Remy N, Scott SL (2015). Inferring causal impact using Bayesian structural time-series models. Ann. Appl. Stat..

[CR12] Calder BJ, Malthouse EC, Schaedel U (2009). An experimental study of the relationship between online engagement and advertising effectiveness. J. Interact. Mark..

[CR13] Deng, T., Ekachai, D., Pokrywczynski, J.: Global COVID-19 advertisements: use of informational, transformational and narrative advertising strategies. Health Commun. 1–9 (2020). 10.1080/10410236.2020.1859725 10.1080/10410236.2020.1859725 33349050

[CR14] Diebolt J, Robert CP (1994). Estimation of finite mixture distributions through bayesian sampling. J. Roy. Stat. Soc.: Ser. B (Methodol.).

[CR15] Fuxman L, Elifoglu IH, Cn Chao (2014). Digital advertising: a more effective way to promote businesses’ products. J. Bus. Admin. Res..

[CR16] Gangadharbatla H (2021). Covid-19 and advertising: the case for a paradigm shift. J. Curr. Issues Res. Advert..

[CR17] Georgieva-Trifonova T, Dechev M (2021). Applying text mining methods to extracting information from news articles. IOP Conf. Ser. Mater. Sci. Eng..

[CR18] Gong S, Zhang J, Zhao P (2017). Tweeting as a marketing tool: a field experiment in the TV industry. J. Mark. Res..

[CR19] Hida, R., Takeishi, N., Yairi, T., et al.: Dynamic and static topic model for analyzing time-series document collections (2018). arXiv:1805.02203

[CR20] Jähnichen P, Wenzel F, Kloft M (2018). Scalable generalized dynamic topic models. Int. Conf. Artif. Intell. Stat. AISTATS.

[CR21] Kantar: COVID-19 barometer: consumer attitudes, media habits and expectations (2022). https://www.kantar.com/inspiration/coronavirus, accessed: 2022-01-21

[CR22] Keller, K.: Strategic Brand Management: Building, Measuring, and Managing Brand Equity, 4th edn. Pearson (2013)

[CR23] Lee YJ, Park JY (2018). Identification of future signal based on the quantitative and qualitative text mining: a case study on ethical issues in artificial intelligence. Qual. Quan..

[CR24] Liu YC, Kuo RL, Shih SR (2020). COVID-19: the first documented coronavirus pandemic in history. Biomed. J..

[CR25] Manning CD, Schütze H (1999). Foundations of Statistical Natural Language Processing.

[CR26] Manning CD, Raghavan P, Schütze H (2008). Introduction to Information Retrieval.

[CR27] Mottl, D.: GetOldTweets3 0.0.11 Python 3 library (2019). https://pypi.org/project/GetOldTweets3/ (Accessed: 19 Jan 2022)

[CR28] Murphy KP (2013). Machine Learning: A Probabilistic Perspective.

[CR29] Nguyen, E.: Text mining and network analysis of digital libraries in R. In: Data Mining Applications with R, pp. 95–115. Elsevier (2014). 10.1016/B978-0-12-411511-8.00004-9

[CR30] Nigam K, Mccallum AK, Thrun S (2000). Text classification from labeled and unlabeled documents using EM. Mach. Learn..

[CR31] Peng J, Agarwal A, Hosanagar K (2018). Network overlap and content sharing on social media platforms. J. Mark. Res..

[CR32] Plummer S, Pati D, Bhattacharya A (2020). Dynamics of coordinate ascent variational inference: a case study in 2D Ising models. Entropy.

[CR33] R Core Team: R: A Language and Environment for Statistical Computing. R Foundation for Statistical Computing, Vienna, Austria (2021). https://www.R-project.org/

[CR34] Robert CP (2007). The Bayesian Choice. Springer Texts in Statistics.

[CR35] Romaniuk J, Sharp B, Paech S (2004). Brand and advertising awareness: a replication and extension of a known empirical generalisation. Australas. Mark. J..

[CR36] Salton G, Wong A, Yang CS (1975). A vector space model for automatic indexing. Commun. ACM.

[CR37] Schweidel DA, Moe WW (2014). Listening in on social media: a joint model of sentiment and venue format choice. J. Mark. Res..

[CR38] Sebastiani F (2002). Machine learning in automated text categorization. ACM Comput. Surv..

[CR39] Silge, J., Robinson, D.: tidytext: text mining and analysis using tidy data principles in r. JOSS **1**(3) (2016). 10.21105/joss.00037

[CR40] Stephen AT, Toubia O (2010). Deriving value from social commerce networks. J. Mark. Res..

[CR41] Tandel, S.S., Jamadar, A., Dudugu, S.: A survey on text mining techniques. in: 2019 5th international conference on advanced computing & communication systems (ICACCS). IEEE, pp. 1022–1026 (2019). 10.1109/ICACCS.2019.8728547

[CR42] Taylor C (2020). Advertising and COVID-19. Int. J. Advert..

[CR43] Tran, M.N., Nguyen, T.N., Dao, V.H.: A practical tutorial on Variational Bayes (2021). arXiv:2103.01327

[CR44] Vaughan K, Beal V, Romaniuk J (2016). Can brand users really remember advertising more than nonusers? Testing an empirical generalization across six advertising awareness measures. J. Advert. Res..

[CR45] Wickham H, Averick M, Bryan J (2019). Welcome to the tidyverse. J. Open Source Softw..

[CR46] Wilbur WJ, Kim W (2009). The ineffectiveness of within-document term frequency in text classification. Inf. Retrieval.

[CR47] Xun J (2015). Return on web site visit duration: applying web analytics data. J. Direct Data Digit. Mark. Pract..

[CR48] Zhang C, Butepage J, Kjellstrom H (2019). Advances in variational inference. IEEE Trans. Pattern Anal. Mach. Intell..

